# Natural hybridization between *Phyllagathis* and *Sporoxeia* species produces a hybrid without reproductive organs

**DOI:** 10.1371/journal.pone.0227625

**Published:** 2020-01-08

**Authors:** Shuaixi Zhou, Shuheng Ni, Jinhong Dai, Qiujie Zhou, Renchao Zhou, Ying Liu

**Affiliations:** State Key Laboratory of Biocontrol and Guangdong Key Laboratory of Plant Resources, School of Life Sciences, Sun Yat-sen University, Guangzhou, Guangdong, China; University of Florida, UNITED STATES

## Abstract

Natural hybridization plays important roles in plant evolution and speciation. In this study, we sequenced ribosomal internal transcribed spacer (nrITS), four low-copy nuclear genes (*Dbr1*, *SOS4a*, *SOS4b* and *PCRF1*) and the chloroplast intergenic spacer *trnV-trnM* to test the hypothesis of hybridization between two species of *Phyllagathis* and *Sporoxeia* (Sonerileae/Dissochaeteae, Melastomataceae). Our results provided compelling evidence for the hybridization hypothesis. All hybrid individuals sampled were first-generation hybrids. The failure of flower production in the F1 hybrid individuals may work as the barrier preventing later-generation hybridization or backcross. Analysis of the chloroplast *trnV-trnM* sequences showed that the hybridization is bidirectional with *S*. *petelotii* as the major maternal parent. Several factors, such as sympatry, similar habitat preference, overlapping flowering season and shared pollinators, might have contributed to this hybridization event. The "intergeneric" hybridization reported in this study suggests close relationship between *P*. *longicalcarata* and *S*. *petelotii*.

## Introduction

Hybridization plays an important role in evolution and speciation [1–4], leading to various evolutionary consequences such as transfer of adaptive traits between species [3, 5–7], evolution of new lineages [4, 8], reinforcement of reproductive barriers [9, 10], species refusion [11, 12] and even extinction of rare species [13, 14]. Hybridization is widespread in flowering plants [15–17]. According to [18], at least 25% of plant species are involved in hybridization and potential introgression with other species. Nevertheless, hybrids are not uniformly distributed across different groups of vascular plants [15, 19, 20] and hybridization of species within genera are far more common than those between genera. In this paper, we report a case of hybridization within Myrtales, an order with low hybridization propensity [20].

**Fig 1:**
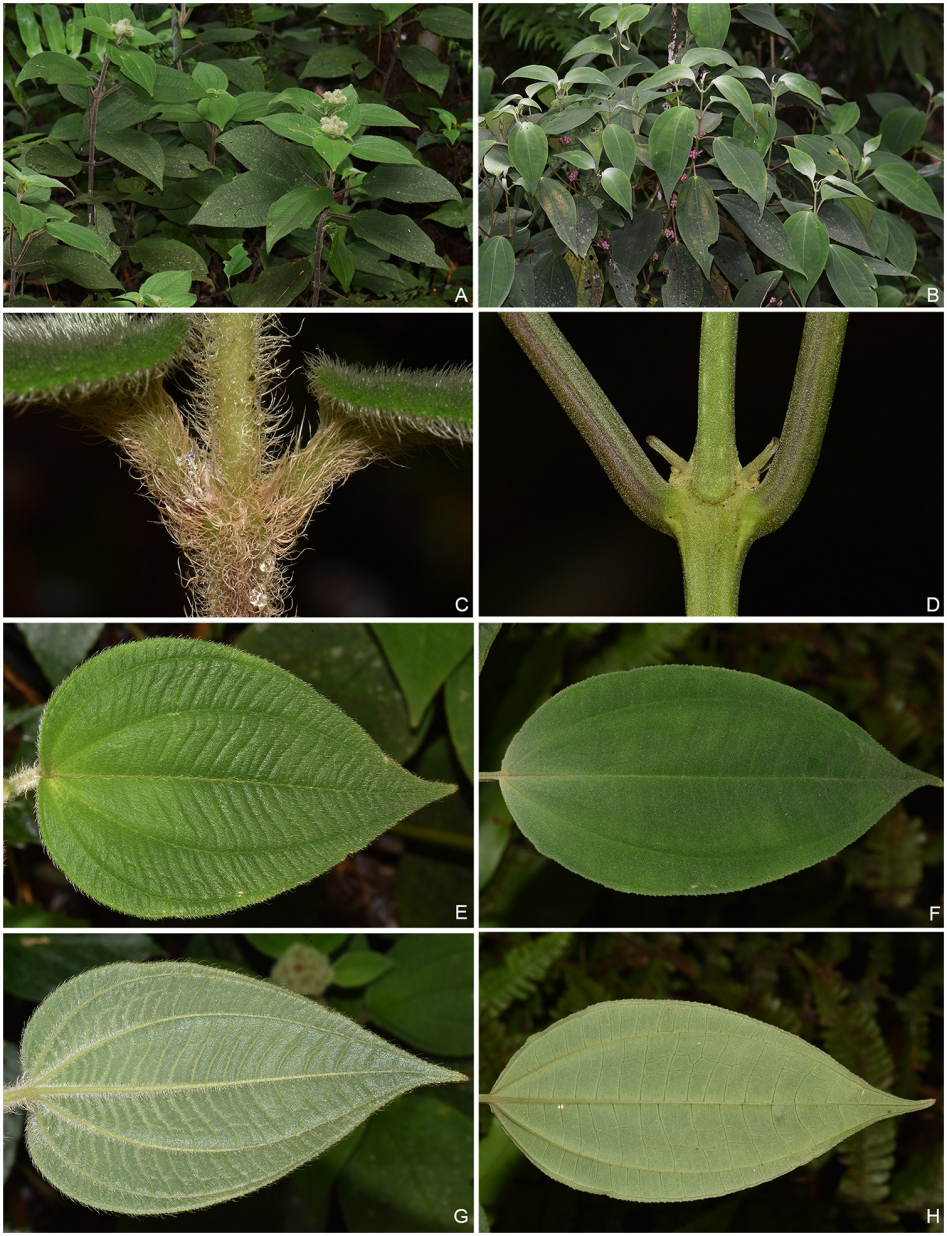
Morphological comparison between *Phyllagathis longicalcarata* (A, C, E, G) and *Sporoxeia petelotii* (B, D, F, H). A–B: Habit. C–D: Stem. E–F: Adaxial surface of leaf blade. G–H: Abaxial surface of leaf blade.

**Fig 2:**
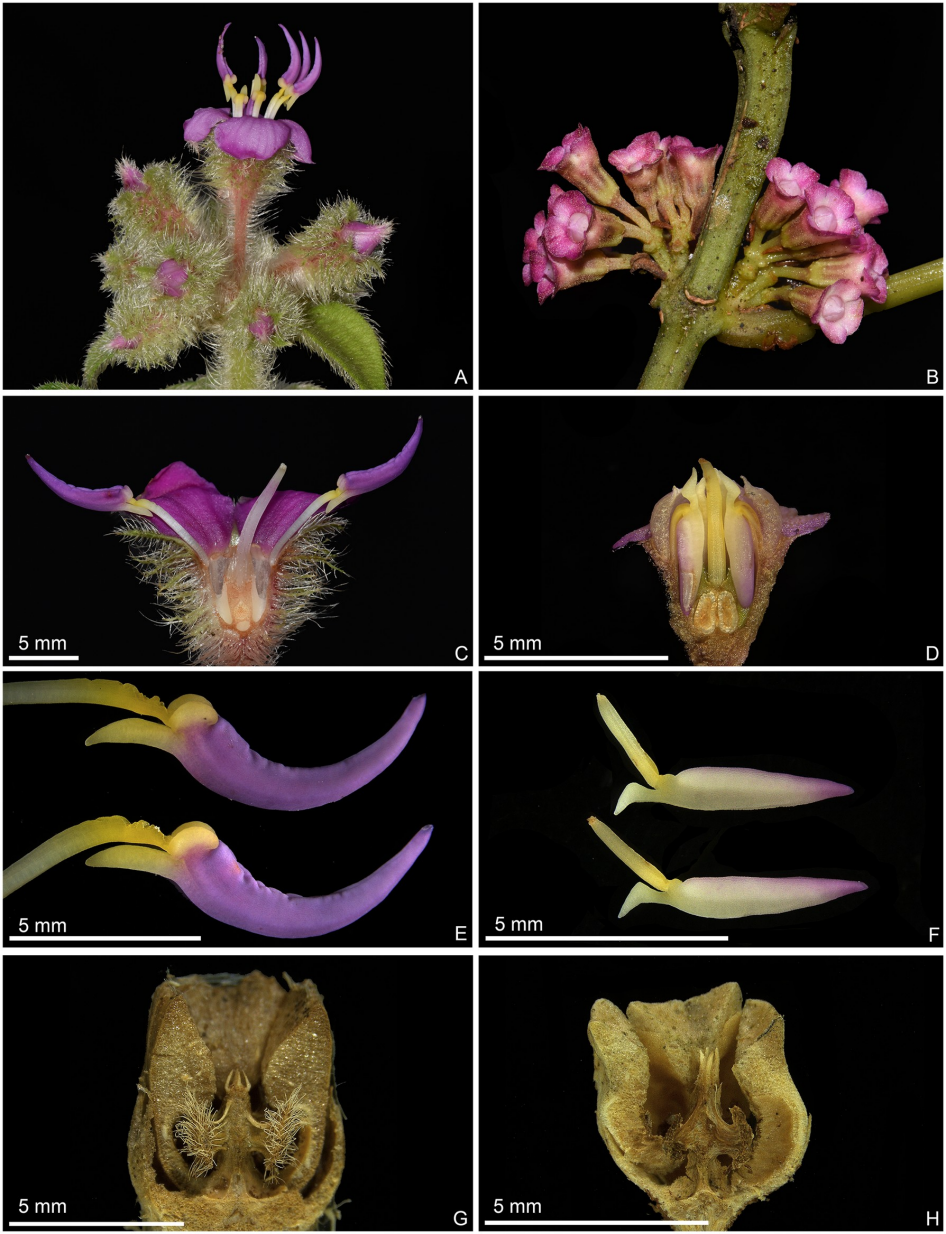
Morphological comparison between *Phyllagathis longicalcarata* (A, C, E, G) and *Sporoxeia petelotii* (B, D, F, H). A–B: Inflorescence. C–D: Longitudinal section of flower. E–F: Stamens. G–H: Longitudinal section of old fruit.

*Phyllagathis longicalcarata* C. Hansen and *Sporoxeia petelotii* (Merr.) C. Hansen are two members of Sonerileae/Dissochaeteae, Melastomataceae (Myrtales). Both species are distributed in southeastern Yunnan, China and northern Vietnam and they co-occur in the forest of Fenshuiling National Nature Reserve in Jinping County, southeastern Yunnan. Field observations show that *P*. *longicalcarata* and *S*. *petelotii* are morphologically distinct from each other, differing markedly in indumentum, number of foliar veins, architecture of inflorescence, flower size, and morphology of calyx and placentas. As shown in Figs 1 and 2, the stems, leaves, inflorescence and calyx lobes of *P*. *longicalcarata* are densely hirsute with long, spreading, multiseriate trichomes, while those of *S*. *petelotii* are sparsely pubescent with very short, appressed uniseriate trichomes and glabrescent at maturity. The two species bear 7 and 5 veins, respectively. The inflorescences of *P*. *longicalcarata* are terminal (Figs 1A and 2A), while those of *S*. *petelotii* are axillary or present at petiole scars on leafless branchlets (Figs 1B and 2B), which is characteristic of *Sporoxeia* W. W. Smith. In addition, *P*. *longicalcarata* has larger flowers, hirsute hypanthium with spreading, branched trichomes, triangular-ovate, green calyx lobes, and thready placentas, while *S*. *petelotii* has smaller flowers, glabrescent hypanthium, broadly ovate, pink calyx lobes, and unthready placentas (Fig 2).

**Fig 3:**
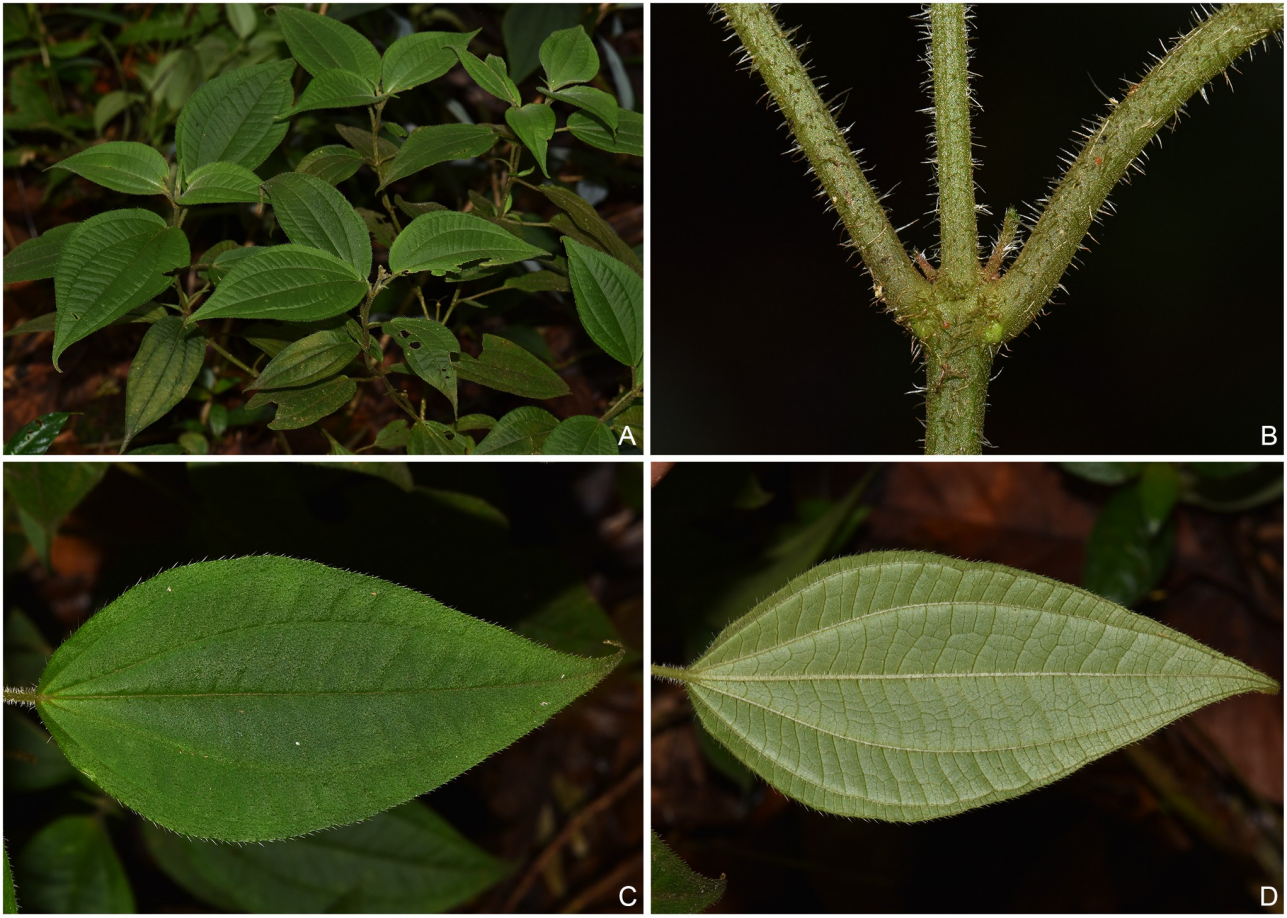
Putative hybrid between *Phyllagathis longicalcarata* and *Sporoxeia petelotii*. A: Habit. B: Stem. C: Adaxial surface of leaf blade. D: Abaxial surface of leaf blade.

During a field survey in 2018, we encountered a group of plant individuals (> 20) in Ma-an-di, Jinping County, southeastern Yunnan, growing alongside with *P*. *longicalcarata* and *S*. *petelotii* at the forest margin between 1800 and 2100 m in elevation. Their stems and leaves are sparsely villous with spreading, multiseriate trichomes, and the leaf blade bears 5 veins, or 7 veins with the outermost pair inconspicuous (Fig 3), all of which are intermediate between *P*. *longicalcarata* and *S*. *petelotii* (densely hirsute, 7 veins vs. sparsely pubescent or glabrescent, 5 veins) (Fig 1). However, we have not found any reproductive organs in these individuals. Based on the above data, we hypothesize that the individuals in question represent hybrids between *P*. *longicalcarata* and *S*. *petelotii*.

In this study, we sequence nuclear ribosomal internal transcribed spacer (nrITS), four low-copy nuclear genes (*Dbr1*, *SOS4a*, *SOS4b* and *PCRF1*), and one chloroplast intergenic spacer (*trnV-trnM*) to (1) test the hybrid origin of the morphologically intermediate individuals, (2) examine the extent of hybridization when the hybrid origin is verified, and (3) determine the direction of the hybridization event.

## Materials and methods

### Ethics statement

Sample collection from the protected area is approved by the Management Bureau of Fenshuiling National Nature Reserve, Jinping, China.

### Sampling

We sampled one population each of *P*. *longicalcarata* (20 individuals), *S*. *petelotii* (20 individuals) and the putative hybrid (14 individuals) from Ma-an-di, Jinping County, southeastern Yunnan, China. All samples were collected along the trailside between 1800 and 2100 m a.s.l. where they co-occur. Fresh leaves were sampled for subsequent DNA extraction.

### DNA extraction, polymerase chain reaction and sequencing

Genomic DNA was extracted from fresh leaf samples using Magen Plant DNA Extraction Kit (Magen, Guangzhou, China). Nuclear ribosomal ITS region, *Dbr1*, two copies of *SOS4* (*SOS4a*, *SOS4b*), *PCRF1* and the chloroplast intergenic spacer (*trnV-trnM*) were amplified and sequenced using the primers listed in Table 1. We purified the PCR products using the Pearl Gel Extraction Kit (Pearl Bio-tech, Guangzhou, China) and then directly sequenced them on an ABI 3730 DNA automated sequencer with the BigDye chemistry (Applied Biosystems, Foster City, CA, USA). For sequences with more than one polymorphic site and insertion/deletion polymorphisms, PCR products were cloned and sequenced to phase the haplotypes. We conducted ligation reactions with a pMD18-T&A Cloning Kit (Takara, Dalian, China) and selected eight positive colonies for each individual for sequencing. The sequences of all haplotypes were deposited in GenBank under accession numbers MN380832–MN380893.

**Table 1:** Sequences of the six pairs of primers used in this study.

Marker	Primer	Sequence	Source
nrITS	ITS4	TCCTCCGCTTATTGATATGC	White et al. 1990
	ITS5	GGAAGTAAAAGTCGTAACAAGG	
*Dbr1*	lde-F	CGTCTTCATCGGTGGAAACC	designed based on our transcriptome sequences (unpublished data)
	lde-R	ACGGACGTGATAAACAGACCT	
*SOS4a*	sos4a-F	TCGCAGACACCTATACACCAG	adapted from Reginato et al. 2016
	sos4a-R	GCTCGAAGCGAACGATTTAC	
*SOS4b*	sos4b-F	ACATAGCACAACAAGAAGCAGC	adapted from Reginato et al. 2016
	sos4a-R	CTGCTGCTTACAATACTTTGTTTC	
*PCRF1*	PCRF1-F	GCAATTCTGCCTCAGTCTAGTG	adapted from Reginato et al. 2016
	PCRF1-R	CGATCGTATTAATTGAGGACCA	
*trnV-trnM*	M-TRN-V	GCTATACGGGCTCGAACC	Hwang et al. 2000
	M-TRN-M	TACCTACTATTGGATTTGAACC	

### Data analyses

Sequences obtained were aligned using SeqMan v.7.1.0 (DNASTAR Inc., Madison, WI) and manually checked. We used DnaSP v. 6 [21] to summarize the haplotypes for each gene. The haplotype network for each gene was then constructed using Network v. 5.0 (www.fluxus-engineering.com) with the median-joining algorithm [22].

## Results

Sequence analyses for each gene were presented below. All differentially fixed nucleotide substitutions and indels were summarized in Tables A–F in S1 File. Sequence alignment was provided in S1 Alignment.

### nrITS

**Fig 4:**
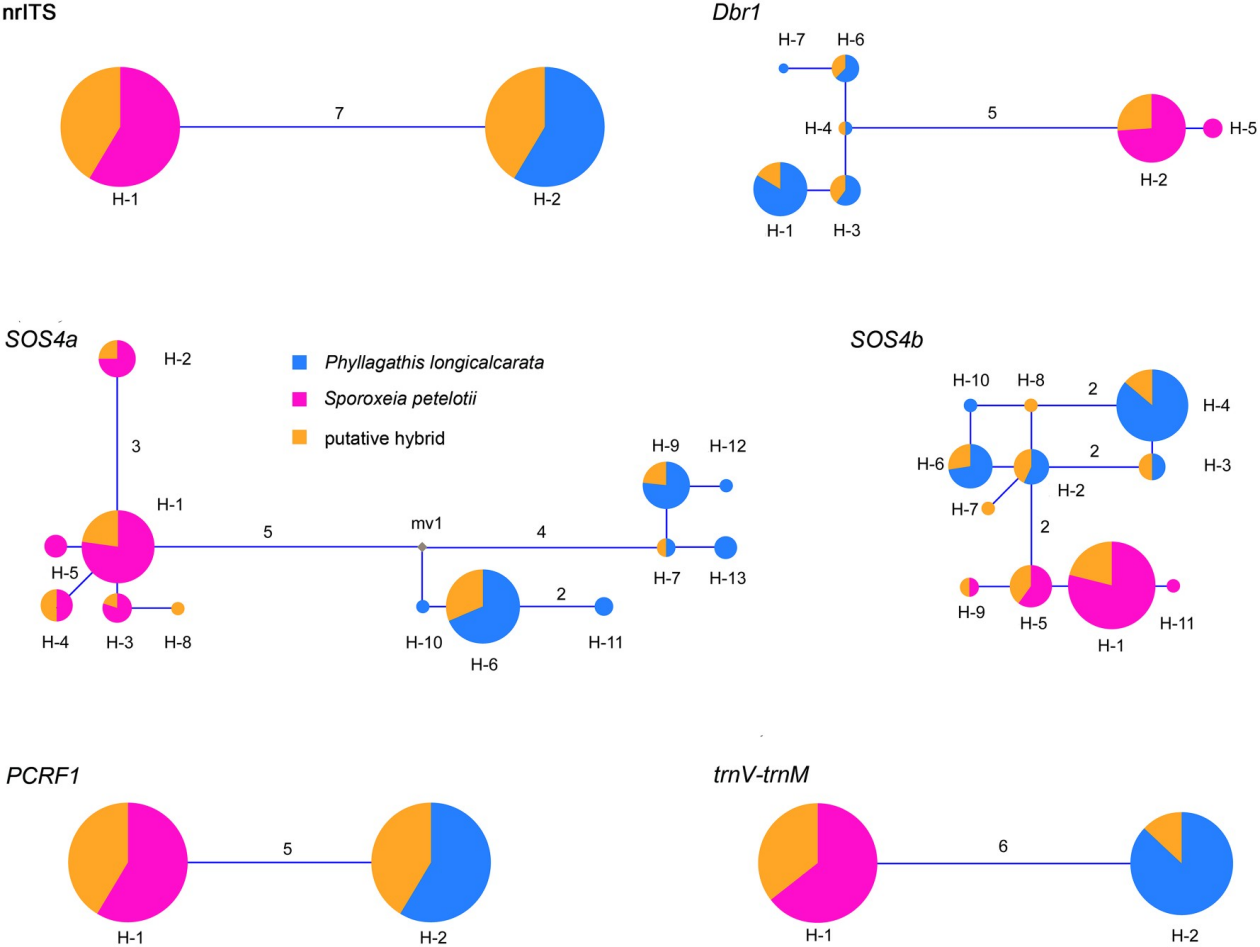
Median-joining networks of nrITS, four nuclear genes (*Dbr1*, *SOS4a*, *SOS4b* and *PCRF1*), and one chloroplast intergenic spacer (*trnV-trnM*) of *Phyllagathis longicalcarata* (in blue), *Sporoxeia petelotii* (in red) and the putative hybrid (in yellow). The numbers on the connecting lines of haplotypes represents the number of mutational steps between them, while those without numbers represent one mutational step. The size of pie-charts is proportional to the frequency for each haplotype.

The aligned nrITS sequences from *P*. *longicalcarata*, *S*. *petelotii* and their putative hybrid was 657 bp in length. No intraspecific polymorphism was detected for *P*. *longicalcarata* and *S*. *petelotii*. We found seven differentially fixed nucleotide substitutions between *P*. *longicalcarata* and *S*. *petelotii*. For the putative hybrid, all individuals showed chromatogram peak additivity at all these fixed sites. Two haplotypes were detected for each individual of the putative hybrid, matching with that of *P*. *longicalcarata* and *S*. *petelotii*, respectively (Fig 4).

### Dbr1

The length of the partial *Dbr1* gene was 505 bp after alignment. Two differentially fixed nucleotide substitutions and two differentially fixed indels were detected between *P*. *longicalcarata* and *S*. *petelotii*, with the putative hybrid showing chromatogram additivity at all these sites. We detected five haplotypes in *P*. *longicalcarata* and two in *S*. *petelotii*. There were five haplotypes in the putative hybrid, four of which were shared with *P*. *longicalcarata* and one was shared with *S*. *petelotii* (Fig 4).

### SOS4a

After sequence alignment, the partial *SOS4a* gene was 529 bp long. There were four fixed nucleotide substitutions and one fixed indel between *P*. *longicalcarata* and *S*. *petelotii*, with the putative hybrid showing chromatogram peak additivity at all these sites. We detected seven haplotypes in *P*. *longicalcarata* and five in *S*. *petelotii*. Of the eight haplotypes in the putative hybrid, three and four were shared with *P*. *longicalcarata* and *S*. *petelotii*, respectively, and one was unique to itself (Fig 4).

### SOS4b

The length of the partial *SOS4b* gene was 263 bp. Two fixed nucleotide substitutions were detected between *P*. *longicalcarata* and *S*. *petelotii*, with the putative hybrid showing chromatogram additivity at each of the two sites. Five haplotypes in *P*. *longicalcarata* and four in *S*. *petelotii* were detected. Of the nine haplotypes in the putative hybrid, four and three were shared with *P*. *longicalcarata* and *S*. *petelotii*, respectively, and the remaining two were unique to itself (Fig 4).

### PCRF1

The aligned sequence of the partial *PCRF1* gene was 271 bp in length. Four fixed nucleotide substitutions and one fixed indel were observed between *P*. *longicalcarata* and *S*. *petelotii*, with the putative hybrid showing chromatogram additivity at all these sites. No intraspecific polymorphism was detected for *P*. *longicalcarata* and *S*. *petelotii*. Again, two haplotypes were detected for each individual of the putative hybrid, matching with that of *P*. *longicalcarata* and *S*. *petelotii*, respectively (Fig 4).

### trnV-trnM

The length of the chloroplast *trnV-trnM* was 815 bp for the three taxa. No intraspecific sequence variation was detected in *P*. *longicalcarata* and *S*. *petelotii*, whereas six fixed nucleotide substitutions were found between them. Of the 14 individuals of the putative hybrid sampled, 11 had the same sequence as *S*. *petelotii* and the remaining three had identical sequence to *P*. *longicalcarata* (Fig 4).

## Discussion

### Molecular evidence for natural hybridization between *P*. *longicalcarata* and *S*. *petelotii*

The putative hybrid grows sympatrically with *P*. *longicalcarata* and *S*. *petelotii* and shows obvious morphological intermediacy between the two species. In this study, we use sequence data of nrITS, *Dbr1*, *SOS4a*, *SOS4b*, *PCRF1*, and chloroplast *trnV-trnM* to test the hybrid origin hypothesis of the putative hybrid. Multiple individuals of *P*. *longicalcarata* (20), *S*. *petelotii* (20) and the putative hybrid (14) were analyzed. We detected multiple differentially fixed nucleotide substitutions and indels at the five nuclear genes and chloroplast *trnV-trnM*, indicating that *P*. *longicalcarata* and *S*. *petelotii* were well separated at these regions. For the biparentally inherited nuclear genes, all individuals of the putative hybrid sampled showed perfect chromatogram additivity at all the fixed sites, while for the uniparentally inherited chloroplast *trnV-trnM*, all 14 individuals shared identical sequence with either *P*. *longicalcarata* or *S*. *petelotii*. Network analysis showed that all but three haplotypes of the putative hybrid were shared with its putative parental species at these six markers. The three unique haplotypes of the putative hybrid, one at the *SOS4a* gene and two at the *SOS4b* gene, have only one mutational step from those of its putative parental species and might come from unsampled polymorphisms of the parental species. Our results provide compelling evidence for the natural hybridization between *P*. *longicalcarata* and *S*. *petelotii* and the hybrid origin of the tested individuals.

### The extent of hybridization and hybridization direction

Introgression is one of the common outcomes of natural hybridization and it may lead to increase of genetic diversity, adaptive gene transfer or extinction of rare species. However, in our case, every hybrid individual sampled is heterozygous at all five nuclear markers, with two haplotypes matching with its parental species, suggesting that all hybrid individuals sampled were first-generation (F1) hybrids, with no sign of later-generation hybrids or backcross progeny. We also carried out a field survey to investigate the flowering and fruiting of the hybrid, focusing only on the potential mature individuals (50 to120 cm in height). However, neither flowers nor fruits were observed on these individuals from April 2018 to October 2019. The two parental species, *P*. *longicalcarata* and *S*. *petelotii*, are quite different in terms of inflorescence position (terminal vs. axillary, often on old wood). It is probable that hybridization between the two species with different inflorescence positions has caused complex gene interaction on inflorescence development in the hybrids, and thus the failure of flower production. This should, in turn, work as the barrier preventing later-generation hybridization and backcrossing to either parental species for the hybrids. Since all these hybrid individuals are F1s, the direction of hybridization can be inferred by chloroplast markers. Analysis of the chloroplast *trnV-trnM* showed that 11 individuals of the hybrid shared the same chlorotype with *S*. *petelotii* and the remaining three with *P*. *longicalcarata*. Therefore, the hybridization between *P*. *longicalcarata* and *S*. *petelotii* is bidirectional with the latter species as the major maternal parent.

### Factors contributing to natural hybridization between *P*. *longicalcarata* and *S*. *petelotii*

Several factors might have contributed to natural intergeneric hybridization between *P*. *longicalcarata* and *S*. *petelotii*. First, the two species were both endemic to northern Vietnam and Jinping county, southeastern Yunnan, China. They prefer similar shady and moist habitats, often occurring along small trails under forest with their elevation range overlapping above 1800 m. Field observation showed that at least in Jinping, the onset of flowering in both species started in June, with *P*. *longicalcarata* flowering only about one week earlier than *S*. *petelotii*. Shared pollinators are also possible, as the flowers of *P*. *longicalcarata* and *S*. *petelotii* are ca. 2 cm and 1 cm in diameter respectively, and therefore both can be visited by medium to small size bees.

### Taxonomic implications

Cases of natural hybridization had been reported for some genera in Melastomataceae [23–26]. Most of them (if not all) are interspecific hybridizations and many are from the tribe Melastomateae. This study represents the first report of natural hybridization between species of two traditional genera in Sonerileae/Dissochaeteae. *Phyllagathis* and *Sporoxeia* are morphologically distinct in inflorescence position (terminal vs. axillary). However, previous phylogenetic analyses, together with reconstruction of morphological characters [27], clearly indicated that many of the characters traditionally used in generic delimitation are highly homoplasious and extensive taxonomic reshuffling at the generic level would be needed to achieve monophyly. The most recent study clearly shows the taxonomic complexity and polyphyly of *Phyllagathis* and *Sporoxeia* and that the relationship between the parental species, *P*. *longicalcarata* and *S*. *petelotii*, is much closer than expected [28]. Both species appear to be nested in a mixed clade with *P*. *hispidissima* (C. Chen) C. Chen and other *Sporoxeia* species [28]. The "intergeneric" hybridization reported in this study provides another line of evidence for the close relationship between *P*. *longicalcarata* and *S*. *petelotii*, urging for more comprehensive phylogenetic studies aimed at clarifying the delimitation of *Phyllagathis* and *Sporoxeia*, in addition to many other taxa within the Sonerileae/Dissochaeteae.

## Supporting information

S1 Alignment Sequence alignment of nrITS, *Dbr1*, *SOS4a*, *SOS4b*, *PCRF1* and *trnV-trnM*. (TXT)

S1 File Differentially fixed nucleotide substitutions and indels detected in nrITS (Table A), *Dbr1* (Table B), *SOS4a* (Table C), *SOS4b* (Table D), *PCRF1* (Table E) and *trnV-trnM* (Table F). (DOCX)
