# Supplementary material for: Natural hybridization between Phyllagathis and Sporoxeia species produces a hybrid without reproductive organs
Source: PLoS One. 2020 Jan 8;15(1):e0227625. doi: 10.1371/journal.pone.0227625 (PMC6949006; doi:10.1371/journal.pone.0227625)
Supplement: S1 File — (DOCX) [file pone.0227625.s002.docx]

Table A

| Marker | nrITS |  |  |  |  |  |  |
| --- | --- | --- | --- | --- | --- | --- | --- |
| Taxon | Variable site | |  |  |  |  |  |
|  | 181 | 182 | 402 | 528 | 579 | 587 | 644 |
| *S. petelotii* | G | A | A | G | T | A | G |
| *P. longicalcarata* | A | T | G | A | C | C | A |
| Putative hybrid | R | W | R | R | Y | M | R |

Table B

| Marker | *Dbr1* |  |  |  |
| --- | --- | --- | --- | --- |
| Taxon | Variable site |  |  |  |
|  | 119 | 122-123 | 131 | 414 |
| *S*. *petelotii* | - | -- | T | G |
| *P. longicalcarata* | C | TG | G | A |
| Putative hybrid | C/- | TG/-- | K | R |

Table C

| Marker | *SOS4a* |  |  |  |  |
| --- | --- | --- | --- | --- | --- |
| Taxon | Variable site | |  |  |  |
|  | 63-66 | 157 | 335 | 448 | 495 |
| *S*. *petelotii* | ---- | C | A | A | A |
| *P. longicalcarata* | TGGT | A | G | G | G |
| Putative hybrid | TGGT/---- | M | R | R | R |

Table D

| Marker | *SOS4b* |  |
| --- | --- | --- |
| Taxon | Variable site |  |
|  | 28 | 29 |
| *S*. *petelotii* | A | A |
| *P. longicalcarata* | T | G |
| Putative hybrid | W | R |

Table E

| Marker | *PCRF1* |  |  |  |  |
| --- | --- | --- | --- | --- | --- |
| Taxon | Variable site |  |  |  |  |
|  | 143 | 167 | 249 | 250-254 | 265 |
| *S. petelotii* | G | A | G | ----- | G |
| *P. longicalcarata* | A | G | T | CTCCA | A |
| Putative hybrid | R | R | K | CTCCA/----- | R |

Table F

| Marker | *trnV-trnM* | |  |  |  |  |
| --- | --- | --- | --- | --- | --- | --- |
| Taxon | Variable site | |  |  |  |  |
|  | 156 | 193 | 448 | 527 | 529 | 724 |
| *S*. *petelotii* | A | G | A | G | A | C |
| *P. longicalcarata* | C | C | G | A | C | G |
| Putative hybrid | M | S | R | R | M | S |
